# Development of a simple estimation method of serum caffeine concentration using a point-of-care test kit for urinary caffeine

**DOI:** 10.1007/s11419-024-00692-2

**Published:** 2024-08-27

**Authors:** Kenji Tsujikawa, Yuki Okada, Hiroki Segawa, Tadashi Yamamuro, Kenji Kuwayama, Tatsuyuki Kanamori, Yuko. T. Iwata

**Affiliations:** National Research Institute of Police Science, 6-3-1 Kashiwanoha, Kashiwa, Chiba 277-0882 Japan

**Keywords:** Caffeine, Serum concentration, Point-of-care test kit, Flatbed scanner, ImageJ

## Abstract

**Purpose:**

Serum caffeine concentration is an indicator of caffeine intoxication; however, it is difficult to measure it in most emergency departments. We developed a simple estimation method using a point-of-care test kit for urinary caffeine.

**Methods:**

Caffeine-spiked human serum (100, 50, 25, and 10 µg/mL) was diluted 10-, 20-, 50-, and 100-fold with phosphate-buffered saline and applied to the kit. After 5 min incubation, the kit was scanned by a flatbed scanner and the membrane image was processed with ImageJ.

**Results:**

When the 20-fold diluted serum was applied, serum samples with initial caffeine concentration ≤ 25 and ≥ 50 µg/mL were caffeine-negative and -positive, respectively. When the 100-fold diluted serum was applied, none of the caffeine-spiked serum samples gave positive results. Therefore, we proposed the following test procedure: (i) 20-fold diluted serum was initially tested and (ii) 100-fold diluted serum was additionally tested when the initial result was caffeine positive. Using this procedure, caffeine concentration is expected to be classified into three levels: ≤ 25, > 25– ≤ 100, and > 100 µg/mL, which almost correspond to no or mild, severe, and potentially fatal intoxication, respectively. The test procedure was validated using postmortem heart blood from two cases of fatal caffeine intoxication (caffeine concentration: 276 and 175 µg/mL) and two cases of other intoxication.

**Conclusions:**

Our developed method using point-of-care urinary caffeine test kits enabled simple estimation of serum caffeine concentration.

**Supplementary Information:**

The online version contains supplementary material available at 10.1007/s11419-024-00692-2.

## Introduction

Caffeine (CAF) is a xanthine derivative found in coffee, chocolate, green tea, black tea, etc. Because CAF shows central nervous system stimulating effects, including awakening, tablets and drops of CAF (100–200 mg per dose) are sold as over-the-counter drugs to reduce sleepiness and fatigue in Japan.

Although the normal dosage of CAF is safe [1], its therapeutic-to-toxic ratio is narrow [2]. Toxic symptoms begin appearing with 1 g CAF, 2 g CAF requires hospitalization, and higher doses of CAF can be fatal [3]. Clinical features of CAF intoxication include cardiovascular (e.g., hypertension, tachycardia, bradycardia), gastrointestinal (e.g., nausea, vomiting), psychological and neurological (e.g., agitation, seizure), and metabolic (e.g., hypokalemia, metabolic acidosis) symptoms [3].

Serum CAF concentration is an indicator of CAF intoxication. Generally, ≥ 15 and ≥ 80 µg/mL CAF is toxic and potentially fatal, respectively [4]. According to Yoshizawa et al., 140 µg/mL of serum CAF is a criterion for initiating hemodialysis, an effective treatment for CAF intoxication [5].

CAF intoxication cases have recently increased in Japan. Kamijo et al. have reported 101 cases of CAF intoxication, including 3 deaths [6]. The report endorses that CAF intoxication is not rare and many emergency departments have experienced it. Rapid determination, or at least estimation, of serum CAF concentration in each emergency department will be useful for the diagnosis and treatment of CAF intoxication. However, most emergency departments, especially secondary ones, do not have a system to determine serum CAF concentration by instrumental analysis, such as gas chromatography and high-performance liquid chromatography [7].

To develop a simple method for estimating serum CAF concentration without expensive analytical instruments, we focused on a point-of-care test (POCT) kit for urinary CAF called CAF Rapid Test Cassette (Urine), Hangzhou Alltest Biotech––an immunochromatography kit. This kit is advantageous in terms of low cost (500 JPY per cassette) and sufficient sensitivity (nominal cut-off concentration: 1 µg/mL) for detection of toxic serum CAF concentration.

Similar to other POCT kits for drug screening, the test result is CAF positive when a band at the test zone (hereafter called the CAF band) is not observed. However, it is difficult to judge the presence or absence of a slight band. This difficulty is strengthened by differences in individual interpretation [8], observer’s age [9], and fatigue [10].

Serum samples must be diluted before using the POCT kit for estimating serum CAF concentration. This requires measurement of a small volume of serum sample. However, most emergency departments may not have laboratory apparatus, such as micropipettes, to perform measurements.

In this study, we developed a simple method to estimate serum CAF concentration using the POCT kit for urinary CAF. To mitigate difficulty in judging the presence of the CAF band, we scanned the cassette by a flatbed scanner and the membrane image was processed by ImageJ (National Institutes of Health, USA), an open-source image analysis software. In addition, we evaluated applicability of a serum sampling device (Mitra Clamshell blood collection device, Neoteryx) to measure small volume of serum without a micropipette.

## Materials and methods

### Devices and reagents

A CAF Rapid Test Cassette (Urine), a POCT kit for urinary CAF, was obtained from Hangzhou Alltest Biotech (Hangzhou, China). The nominal cut-off concentration for CAF was 1 µg/mL. The Mitra Clamshell blood collection device (10 µL, hereafter called the Mitra device), a serum sampling device, was obtained from Neoteryx (Torrance, CA).

CAF (anhydrous) was obtained from Wako Pure Chemical (Osaka, Japan). Paraxanthine, theophylline, and theobromine were obtained from Sigma-Aldrich (St. Louis, MO), Enzo Life Sciences (Farmingdale, NY), and Wako Pure Chemical, respectively. Phosphate-buffered saline (PBS) was prepared by dissolving 0.2 g of potassium dihydrogen phosphate, 0.2 g of potassium chloride, 2.9 g of disodium hydrogen phosphate 12-water, and 8.0 g of sodium chloride in water to make 1 L of solution in our laboratory.

### Biological materials and preparation of drug-spiked serum

Blank human serum was obtained from Biopredic International (Saint-Grégoire, France). Four samples from forensic cases (postmortem heart blood) were provided from Educational and Research Center of Legal Medicine, Chiba University. In total, 2/4 samples (A and B) originated from fatal cases of CAF intoxication. CAF concentrations of samples A and B were 276 and 175 µg/mL, respectively. The other two samples (C and D) originated from one case each of fatal acetaminophen intoxication and fatal salicylate intoxication, respectively. All toxicological analyses were performed at Chiba University.

CAF-spiked serum (100, 50, 25, and 10 µg/mL) was prepared by adding 10 µL of 10, 5, 2.5, and 1 mg/mL aqueous CAF to 990 µL of blank human serum, respectively. Paraxanthine-, theophylline-, and theobromine-spiked serum (100 µg/mL each) was prepared by adding 10 µL of their aqueous solutions (10 mg/mL) to 990 µL of blank human serum.

### General test procedure

Drug-spiked sera and forensic case samples were diluted with PBS. PBS was selected to prevent variation of pH, which influences the antigen–antibody reaction [11]. Dilution times were indicated in each experimental subsection. Dilution was performed by micropipettes, unless otherwise specified. According to the package insert, after three drops of the diluted samples were applied to the POCT kit with an attached plastic dropper, the cassette was incubated for 5 min. Then, the cassette was scanned once by a flatbed scanner (CanoScan LiDE400, Canon, Tokyo, Japan) at 400 dpi. Before scanning, the cassette was set to a jig made by corrugated cardboard to fix the position and direction on the scanner (Figure S1).

The scanned images (.jpg file) were processed with ImageJ. The membrane image was cropped, and the image was processed by two processing methods: contrast enhancement (saturated pixels were set to 0.1%) and color inversion. The CAF band was observed on a computer display with the naked eye. Pixel intensities in the original membrane image were converted to a two-dimensional plot using the plot profile function of ImageJ. Peak area corresponding to the CAF band (hereafter called the CAF peak) was calculated.

### Preliminary evaluation of CAF detectability in the serum with the POCT kit

CAF-spiked serum at 50 µg/mL was diluted 10-, 20-, 50-, and 100-fold with PBS. The subsequent procedure was performed according to the “General test procedure” subsection in singlicate.

### Evaluation of reproducibility of scanning

CAF-spiked serum at 50 µg/mL was diluted 100-fold with PBS. The diluted serum was applied to two cassettes to evaluate reproducibility between scanning procedures at the same position and different scanned positions. After 5 min incubation, one cassette was scanned four times at the same position on the scanner and the other cassette was scanned four times at different positions on the scanner. The subsequent procedure was performed according to the “General test procedure” subsection.

### Relationship between initial CAF concentration or dilution times and visible CAF band intensity or CAF peak area

CAF-spiked serum at 100, 50, 25, and 10 µg/mL was diluted 10-, 20-, 50-, and 100-fold with PBS. The subsequent procedure was performed according to the “General test procedure” subsection. The test was performed in triplicate for each dilution times at each serum CAF concentration.

### Performance check using forensic case samples

The forensic samples from cases A–D were diluted 20- and 100-fold with PBS. Diluted samples were tested according to the “General test procedure” subsection in singlicate.

### Evaluation of cross-reactivity to paraxanthine, theophylline, and theobromine

Paraxanthine-, theophylline-, and theobromine-spiked serum (each 100 µg/mL) was diluted 20-fold with PBS. The diluted serum was tested according to the “General test procedure” subsection in triplicate.

### Serum sampling with the *Mitra* device

The Mitra device was dipped in CAF-spiked serum (initial concentration: 100 µg/mL) in a micro test tube. Then, the device was soaked in 190 and 990 µL of PBS in micro test tubes for 5 min, corresponding to 20- and 100-fold dilutions, respectively. As controls, 20- and 100-fold diluted CAF-spiked serum (initial concentration: 100 µg/mL) was prepared using micropipettes. The diluted serum was tested according to the “General test procedure” subsection in triplicate.

## Results

### Preliminary evaluation of CAF detectability in the serum using the POCT kit

**Fig. 1:**
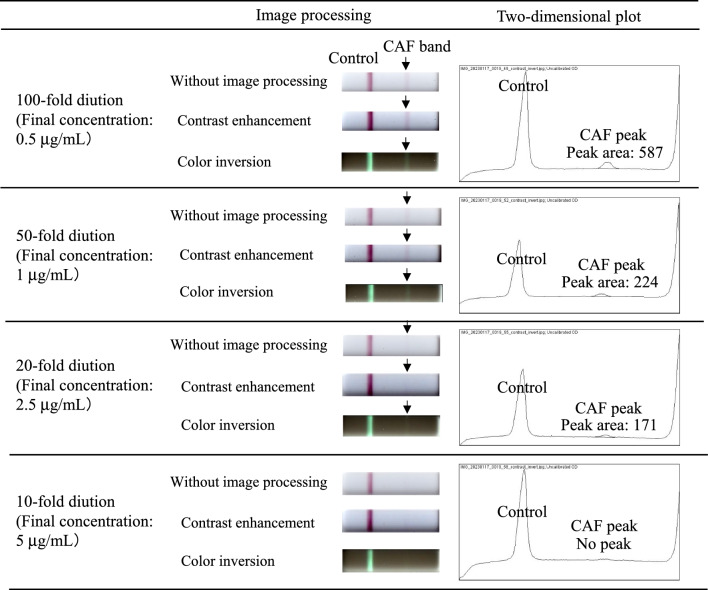
Scanned images with and without image processing, as well as two-dimensional plots for CAF-spiked serum (initial concentration: 50 µg/mL) diluted 10-, 20-, 50-, and 100-fold by PBS

Figure 1 shows scanned images with and without image processing, as well as two-dimensional plots. As the dilution times was larger (that is, the final concentration of CAF was lower), the CAF band was more clearly visible. Visibility of the CAF band was improved by image processing. On the computer monitor, color inversion processing seemed to enhance visibility compared with contrast enhancement processing. After color inversion processing, the CAF band was visible obviously for the 100- and 50-fold diluted samples and slightly for the 20-fold diluted one; however, it was invisible for the tenfold diluted one.

To evaluate the presence of the CAF band objectively, the scanning images were converted to two-dimensional plots. Similar to visibility of the CAF band with the naked eye, the CAF peak was detected for the 100-, 50-, and 20-fold diluted samples, but not for the 10-fold diluted one. As the CAF band was more clearly visible, the CAF peak area was larger.

The preliminary experimental results indicated that (i) serum CAF concentration could be estimated by a combination of the POCT kit and multiple dilutions of the serum sample, (ii) image processing (especially, color inversion) enhanced visibility of the CAF band, and (iii) the two-dimensional plot would be useful for judging the presence or absence of the CAF band objectively. In further experiments, the CAF band was evaluated by visible intensity after color inversion processing and CAF peak area on a two-dimensional plot.

### Evaluation of reproducibility of scanning

A flatbed scanner is an ordinary and inexpensive office equipment, not an analytical instrument. This means that reproducibility among scanning procedures at the same position and among different scanning positions may not be verified before shipping. To use it in place of an analytical instrument, we evaluated reproducibility.

**Fig. 2:**
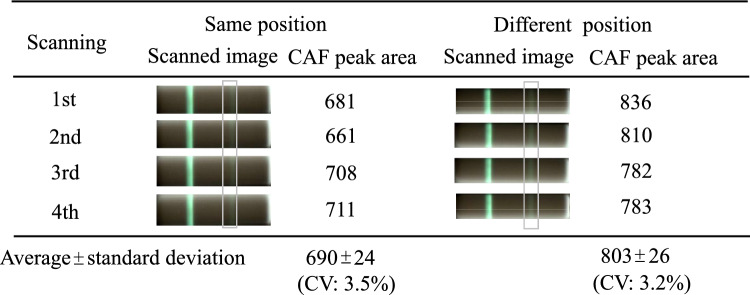
Scanned images after color inversion processing and CAF peak areas on two-dimensional plots for CAF-spiked serum (50 µg/mL) diluted 100-fold by PBS. Scanning was performed at the same position and at different positions on the scanner

Figure 2 shows scanned images after color inversion processing and CAF peak areas on the two-dimensional plots at the same position and at different positions on the scanner. Visible intensities of the CAF band were similar between the scanning procedures at the same position and among different positions. Coefficient of variation of CAF peak area was < 4%. These results indicated that differences among scanning were negligible. In further experiments, maximum four cassettes were simultaneously scanned.

### Relationship between initial CAF concentration or dilution times and visible CAF band intensity or CAF peak area

**Fig. 3:**
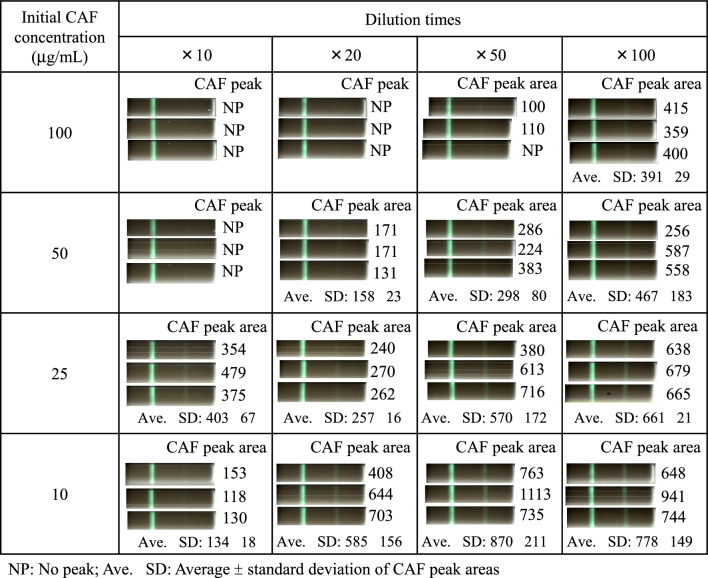
Scanned images after color inversion processing and CAF peak areas for CAF-spiked serum (100, 50, 20, and 10 µg/mL) diluted 10-, 20-, 50-, and 100-fold by PBS

Figure 3 shows scanned images after color inversion processing and CAF peak areas for CAF-spiked serum after dilution with PBS. As final CAF concentration was lower (that is, initial CAF concentration was lower and/or dilution times were higher), visible CAF band intensity tended to be stronger and the CAF peak area tended to be larger.

The relationship between visible CAF band intensity and CAF peak area was as follows: (i) when the CAF band was clearly visible, CAF peak area was ≥ 200; (ii) when the CAF band was slightly visible, the CAF peak was detected but its area was < 200; and (iii) when the CAF band was invisible, the CAF peak was not detected. This relationship indicated that the presence or absence of the CAF band could be judged based on the CAF peak area.

In POCT drug-screening kits based on immunochromatography, the presence of a slight band at the test zone was considered drug negative. However, it may be difficult to practically discriminate between no band and slight band. Therefore, we considered bands not only invisible, but also slightly visible, CAF positive. This is corresponding to that no CAF peaks and CAF peak area < 200 was regarded as CAF positive.

In the clinical setting, CAF concentration in serum samples from a patient suspected of CAF intoxication is unknown. Under this condition, CAF concentration would be estimated from CAF peak areas of diluted serum samples. At 20-fold dilution, initial CAF concentration giving CAF negative and -positive was ≤ 25 and ≥ 50 µg/mL, respectively. At 100-fold dilution, even initial CAF concentration of 100 µg/mL gave CAF-negative results. Conversely, when the test result at 100-fold dilution is CAF-positive, it means that the serum sample contains > 100 µg/mL of CAF.

**Fig. 4:**
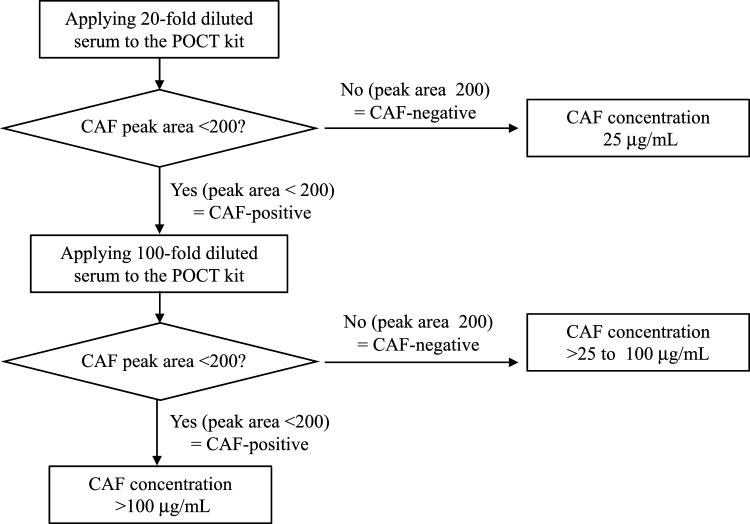
The proposed test procedure for estimating serum CAF concentration

Based on these findings, we proposed a test procedure for estimating serum CAF concentration (Fig. 4). Initially, a 20-fold diluted serum sample is tested. When the test result is CAF negative, CAF concentration is regarded as ≤ 25 µg/mL, corresponding to no or mild intoxication. When the 20-fold diluted serum sample is CAF positive, a 100-fold diluted serum sample is additionally tested. When the test result is CAF negative, CAF concentration is regarded as being within a range between > 25 and ≤ 100 µg/mL, corresponding to severe intoxication. When the test result is CAF positive, CAF concentration is regarded as > 100 µg/mL, corresponding to potentially fatal intoxication.

### Performance check of proposed test procedure using forensic case samples

Because serum samples were not obtained from patients with CAF intoxication, the performance of the proposed test procedure was checked using heart blood samples from four forensic cases (samples A and B, C, and D: CAF, acetaminophen, and salicylate intoxication, respectively). CAF peak areas for the forensic case samples are shown in Table 1. In forensic case samples A and B which were diluted 20- and 100-fold, the CAF peak was either not detected or the peak area was < 200. According to the proposed test procedure, CAF concentrations were judged as > 100 µg/mL. By contrast, in forensic case samples C and D which were diluted 20- and 100-fold, CAF peak areas were ≥ 200. According to the proposed test procedure, their CAF concentrations were judged as ≤ 25 µg/mL. The judgments for all forensic case samples were consisted with the CAF concentration determined by instrumental analysis.

**Table 1:** CAF peak areas for forensic case samples A to D diluted 20- and 100-fold by PBS

Forensic case samples	Dilution		Estimated CAF concentration
	20-fold	100-fold	
A (CAF, 276 µg/mL)	No peak	No peak	> 100 µg/mL
B (CAF, 175 µg/mL)	No peak	139	> 100 µg/mL
C (acetaminophen, no quantification)	666	1429	≤ 25 µg/mL
D (salicylate, 602 µg/mL)	751	1766	≥ 25 µg/mL

Unlike serum samples, whole blood samples turned red on the membrane of the POCT kit. This may lead to a decrease in CAF peak area by increasing background level; however, it was mitigated by ≥ 20-fold dilution. Thus, our test procedure is applicable to not only serum samples, but also whole blood samples.

### Evaluation of cross-reactivity to paraxanthine, theophylline, and theobromine

Paraxanthine, theophylline, and theobromine are mono *N*-desmethyl metabolites of CAF. Considering similarity of chemical structures, CAF-targeted immunoassay may show cross-reactivity to these metabolites. Consistently, an enzyme-linked immunosorbent assay kit for CAF reacted with paraxanthine with a cross-reactivity of 63% [12]. Because there was no information on cross-reactivity to these metabolites in the package insert of the POCT kit, we evaluated it.

To the 20-fold diluted serum whose initial concentration was 100 µg/mL, CAF did not give CAF peak, but paraxanthine, theophylline, and theobromine gave CAF peaks with areas: 1149 ± 153, 671 ± 30, and 241 ± 39 (*n* = 3, average ± standard deviation), respectively. CAF peak area of paraxanthine was larger than that of 20-fold diluted serum (initial CAF concentration: 10 µg/mL). CAF peak areas of theophylline and theobromine were almost equivalent to those of 20-fold diluted serum (initial CAF concentration: 10 and 25 µg/mL, respectively). These results suggest that the reactivity of the POCT kit to paraxanthine, theophylline, and theobromine corresponded to < 1/10, 1/10, and 1/4 of CAF, respectively.

When CAF was overdosed, the highest blood concentration was CAF, followed by paraxanthine, theophylline, and theobromine in this order [13]. Notably, the latter two concentrations were < 1/10 of unchanged CAF [13]. Given that the blood concentration of the three metabolites was lower than that of CAF and showed lower reactivity to the POCT kit than CAF, these metabolites may not influence the estimation of serum CAF concentration with the POCT kit.

### Serum sampling with the *Mitra* device

Our proposed test procedure includes 20- and 100-fold dilution of serum. This requires measurement of a small volume of serum and mixing it with a given volume of PBS in a container. Considering usability in emergency departments, it is preferrable that measurement of serum sample is performed without micropipettes.

To solve this problem, we focused on the Mitra device. The supplier claims that the device may not accurately measure a small volume of blood without a pipette. The authors evaluated the applicability of the Mitra device to the test procedure using CAF-spiked serum of 100 µg/mL.

Next, 100-fold diluted serum prepared by the Mitra device and micropipettes gave CAF peaks with areas 493 ± 87 and 391 ± 29 (*n* = 3, average ± standard deviation), respectively. There was no significant difference in CAF peak area between two serum measurement methods (two-sided Welch’s *t*-test, *p* = 0.19). For the 20-fold diluted serum sample, both serum measurement methods gave no CAF peak. These results show that Mitra device is applicable to the test procedure equivalently to a micropipette.

## Discussion

In this study, we proposed a test procedure to estimate serum CAF concentration by combining the POCT kit, a flatbed scanner, and ImageJ. To apply it to clinical setting, especially secondary emergency departments, the economical perspective is not ignored. The proposed test procedure does not require expensive scientific instrument and only requires the POCT kits and Mitra devices (or micropipettes). Two POCT cassettes and two Mitra devices are required for one test. The cost of two POCT cassettes and two Mitra devices was 1000 JPY and ~ 1000 JPY, respectively. Given that the cost of one POCT drug-screening cassette is 1000–4000 JPY, our proposed test procedure is practical.

Our test procedure may be useful in decreasing unnecessary hospitalization to an intensive care unit (ICU). Severe CAF intoxication is usually treated in an ICU [14]. Considering that ICU hospitalization is expensive (100,000–140,000 JPY per day in Japan), our test procedure will contribute to saving healthcare cost.

## Conclusion

We developed a simple, low-cost, and objective method to estimate serum CAF concentration by combining the POCT kit for urinary CAF, a flatbed scanner, and ImageJ. Our proposed test procedure will contribute to the diagnosis of CAF intoxication in the clinical setting.

## Supplementary Information

Below is the link to the electronic supplementary material.

Supplementary file1 (DOCX 16708 KB)
