# Supplementary figures and images for: Development of a simple estimation method of serum caffeine concentration using a point-of-care test kit for urinary caffeine
Source: Forensic Toxicol. 2024 Aug 27;43(1):155–62. doi: 10.1007/s11419-024-00692-2 (PMC11782425; doi:10.1007/s11419-024-00692-2)

Fig. S1 The jig to fix position and direction of the POCT device on the scanner


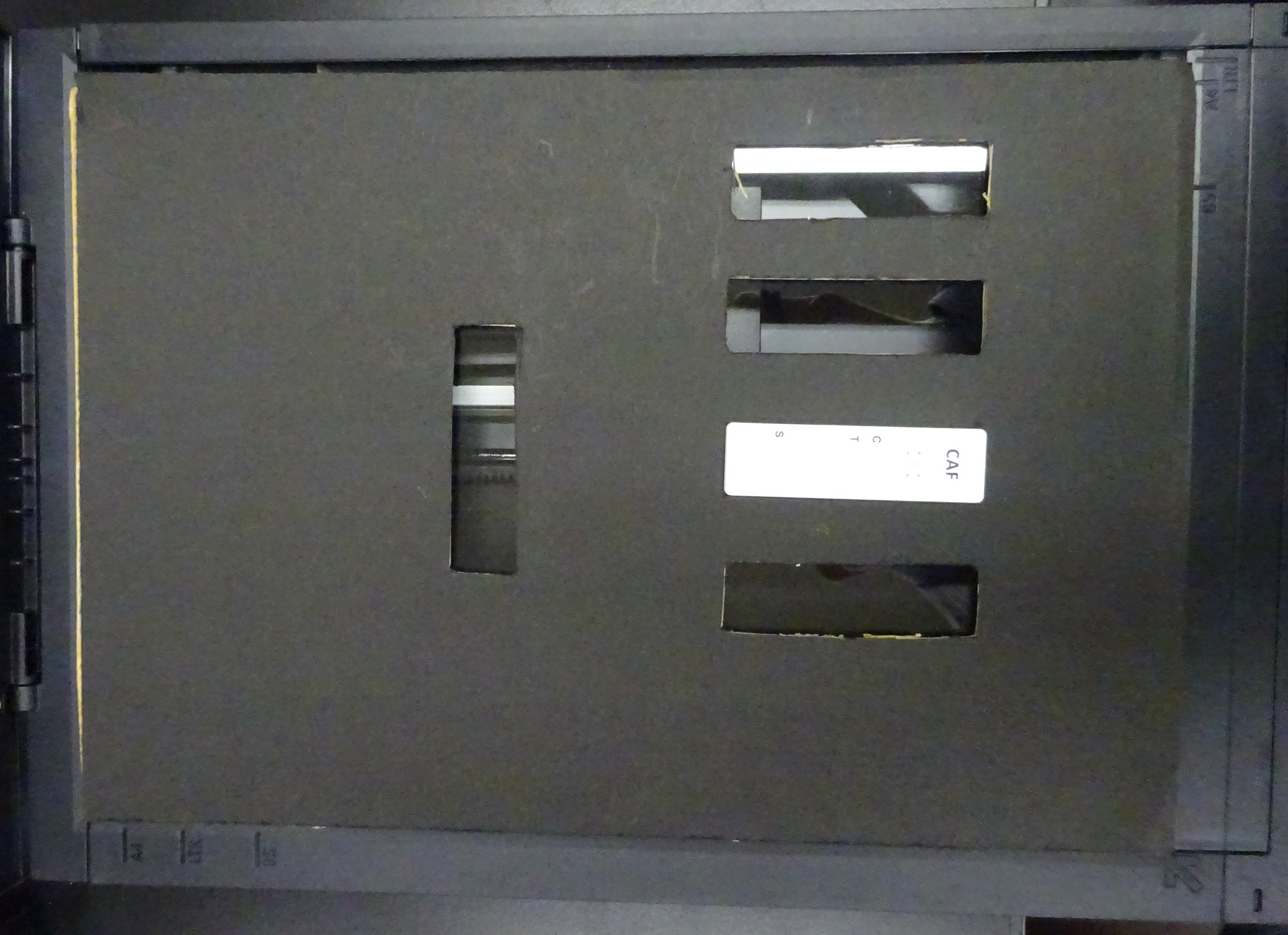

Supplement: Supplementary file 1 — Supplementary file1 (DOCX 16708 KB) [file 11419_2024_692_MOESM1_ESM.docx]
